# Indirect standardization: time to eliminate misleading terminology

**DOI:** 10.1007/s10654-025-01349-z

**Published:** 2026-01-24

**Authors:** Emilio A. L. Gianicolo, Maria Blettner, Andreas Stang

**Affiliations:** 1 Institut für medizinische Biometrie, Informatik und Epidemiologie, Universitätsmedizin der Johannes Gutenberg-Universität Mainz, Mainz, Germany; 2 Institut fur Medizinische Biometrie, Epidemiologie und Informatik, Universitätsmedizin der Johannes Gutenberg-Universität Mainz, Mainz, Germany; 3 Institut für Medizinische Informatik, Biometrie und Epidemiologie (IMIBE) Universitätsklinikum Essen, Essen, Germany

Standardization is one of the oldest statistical techniques for the analysis of epidemiological data. It is largely used to fairly compare populations while accounting for the confounding effect of e.g., different age structures [1, 2]. Most textbooks describe two principal approaches to standardizing rates: the so-called *direct* and the so-called *indirect* method. During the 1980’s, however, Miettinen [3] and Rothman [4] argued that there is actually any distinction between the two methods, and in 2002, Rothman explicitly defined the term “indirect standardization” a misnomer because the two approaches are mathematically equivalent [5].

Both the *direct* and the *indirect* approaches are mathematically identical in that each method computes a weighted sum of age‑specific rates; the only difference lies in the source of the weights: direct standardization uses a set of standard population weights (person-years) [6], whereas *indirect* standardization applies as weights the person-years observed in the exposed population (see supplementary material for the algebra).

As standard population you might choose:

- an external population, for example the Segi-World population [7] or the Old European standard population; [8]
- one of the populations you are comparing or their combination.

When the distribution of person years of an exposed population is assumed as the standard, the ratio of the standardized rates is called standardized incidence ratio (SIR) [6] and this way of standardizing is traditionally called the “indirect” standardization [9–11].

In practice, epidemiological text books [12], and major statistical packages – such as SAS^®^ – continue to employ this terminology: *direct standardization* for the calculation of directly standardized rates and *indirect standardization* for the calculation of the standardized incidence ratio (SIR).

The aim of this research letter is to emphasize again the misleading terms “direct” and “indirect” and show with an example how to use SAS^®^ to obtain SIRs while applying the method of the *direct standardization* in place of the traditional *indirect* formulation producing identical results.

## Methods

We used data on stomach cancer in Cali (Columbia) and North Rhine-Westphalia (NRW, Germany) together with the corresponding population figures for the period 2013–2017 obtained by the International Agency for Research on Cancer [13].

Analyses were performed in SAS^®^ as described below.

- We first calculated the SIR for Cali with NRW serving as the reference population, i.e. the conventional indirect method.
- c) Next, we derived the identical SIR by the direct option of SAS^®^v PROC STDRATE, employing the age-specific person-years from Cali (conceptually the “exposed” group) as the weighting set.

## Results

In the period 2013–2017 crude incidence rates of 21.5 and 22.9 per 100,000 PY were observed in Cali and NRW respectively (Table 1). The crude rate ratio was 0.94 (95% confidence interval: 0.89–0.99). This result is counterintuitive, as one might expect higher incidence rates in a region of the Global South compared to those of the Global North, given the higher prevalence of risk factors for stomach cancer in the former. However, this pattern is due to the different age structures characterizing the two populations, with prevailing higher percentages of younger in Cali than NRW.

- The SIR obtained using the “indirect” option equaled 1.91 (Supplementary material, step a).
- Applying the “direct” option (i.e. using the Cali age‑specific person‑years as the weighting set) produced an identical SIR of 1.91 (Supplementary material, step c).

**Table 1:** Number of stomach cancer cases among males, person years and crude incidence rates in Cali (Columbia) and North Rhine-Westphalia (Germany), 2013–2017

Age group (years)	Cali			North Rhine-Westphalia		
	Number of stomach cancer cases	Person-years	Crude rates per 100.000 person-years	Number of stomach cancer cases	Person-years	Crude rates per 100.000 person-years
0–19	0	1,879,110	0.0	2	8,630,574	0.0
20–39	74	1,886,884	3.9	133	10,853,391	1.2
40–59	351	1,305,887	26.9	1,972	13,425,892	14.7
60–79	585	523,822	111.7	5,505	8,725,513	63.1
≥ 80	206	68,998	298.6	2,343	1,808,240	129.6
Total	1,216	5,664,701	21.5	9,955	43,443,610	22.9

## Discussion

In the present contribution we illustrated that in statistical software like in SAS^®^ “direct” and “indirect” options can be used interchangeably to obtain standardized mortality or incidence ratios. Indeed, students of epidemiology are taught that the direct and the indirect method are mathematically equivalent as long as an internal standard is used. Yet, some statistical software packages used by epidemiologists have perpetuated this *misnomer* in their terminology. The time is now ripe, for us teachers of epidemiology and for developers of statistical software packages, to revise this terminology. We propose that, instead of using the labels “direct” and “indirect”, authors simply describe the source of the weights employed in the standardization. This wording clarifies the analytical choice without relying on ambiguous terms.

## Supplementary Information

Below is the link to the electronic supplementary material.

Supplementary file1 (DOCX 21 kb)

Supplementary file2 (DOCX 55 kb)

## References

[1] Feinleib M, Zarate A. Reconsidering age adjustment procedures; workshop proceedings. Hyattsville, MD. 1992. 1280886

[2] Keiding N. The method of expected number of deaths, 1786-1886-1986. Int Stat Rev. 1987;55(1):1–20. 12179583

[3] Miettinen OS. Theoretical epidemiology: principles of occurrence research in medicine. New York: Wiley; 1985.

[4] Rothman K. Modern epidemiology. 1st Edition. Little Brown & Co.; 1986.

[5] Rothman K. Epidemiology an introduction. 1st edition. Oxford University Press Inc; 2002.

[6] Lash T, VanderWeele T, Haneuse S, Rothman K. Modern epidemiology. 4th Edition. Lippincott Williams and Wilkins; 2021.

[7] Segi M. Cancer mortality for selected sites in 24 countries, no. 4, 1950-57. Sendai, Japan: Tohoku University of Medicine; 1960.

[8] Doll R, Cook P. Summarizing indices for comparison of cancer incidence data. Int J Cancer. 1967;2(3):269–79. 10.1002/ijc.2910020310. 6041994 10.1002/ijc.2910020310

[9] Hennekens C, Buring J. Epidemiology in medicine. Lippincott Williams & Wilkins; 1987.

[10] Szklo M, Nieto F. Epidemiology: Beyond the Basics. Jones & Bartlett Learning; 2004.

[11] Gordis L, Epidemiology. Saunders; 2004 2nd Edition.

[12] Webb P, Bain C, Page A. Essential epidemiology. 5th Edition. Cambridge University Press; 2024.

[13] Bray F, Colombet M, Aitken J, et al. Cancer incidence in five continents. Lyon: International Agency for Research on Cancer; 2023.

