# Supplementary material for: Indirect standardization: time to eliminate misleading terminology
Source: Eur J Epidemiol. 2026 Jan 24;41(4):527–9. doi: 10.1007/s10654-025-01349-z (PMC13331817; doi:10.1007/s10654-025-01349-z)

**Supplementary material**

**Algebra**

Given two populations A and B, the ratio of two standardized rates $R^{A*}$and $R^{B*}$ can be expressed as:

$${RR}_{std}=\frac{R^{A*}}{R^{B*}}=\frac{\sum_{i=1}^{k} {PY}_{i}^{*}\times R_{i}^{A}}{\sum_{i=1}^{k} {PY}_{i}^{*}\times R_{i}^{B}}$$

where $i=1 to k$ indicates the age group;

${PY}_{i}^{*}$ represents the distribution of the person years of a standard population;

$R_{i}^{A}$ and $R_{i}^{B}$represent age specific rates for a given outcome $m_{i}$for the populations A ($R_{i}^{A}=\frac{m_{i}^{A}}{{PY}_{i}^{A}}$) and B ($R_{i}^{A}=\frac{m_{i}^{A}}{{PY}_{i}^{A}}$).

**SAS program.**

**proc** **format**;

value age

**1**="0–19 years" **2**="20–39" **3**="40–59" **4**="60–79" **5**="=>80";

value state

**1**="Cali" **2**="North Rhine-Westphalia (NRW)"; **run**;

**data** pops;

attrib

state label="State"

age label="Age-class"

cases label="Number of cases"

py label="Person-years"

;input state age cases py;

cards;

1 1 0 1879110

1 2 74 1886884

1 3 351 1305887

1 4 585 523822

1 5 206 68998

2 1 2 8630574

2 2 133 10853391

2 3 1972 13425892

2 4 5505 8725513

2 5 2343 1808240

;

**run**;

**data** oldeu;

attrib

age label="Age-group"

w_oldeu label ="Weights of Old EU population"

;input age w_segi;

cards ;

1 0.29

2 0.28

3 0.27

4 0.14

5 0.02

;

**run**;

**data** cali nrw;

set pops;

if state = **1** then output cali;

if state = **2** then output nrw;

**run**;

* a) Direct standardized rates;

**proc** **stdrate** data = pops refdata = oldeu method = direct stat = rate(mult = **100000**) effect = ratio;

population group = state event = cases total = PY;

reference total=w_oldeu; strata age/effect; format state state.;**run**;

Strata Rate Effect Estimates (Rate Multiplier = 100000)

-----------state----------

North

Stratum Rhine-Westphalia Rate 95% Lognormal

Index age Cali (NRW) Ratio Confidence Limits

1 1 0.000 0.023 0.00000 . .

2 2 3.922 1.225 3.20037 2.40854 4.25252

3 3 26.878 14.688 1.82994 1.63353 2.04998

4 4 111.679 63.091 1.77013 1.62551 1.92762

5 5 298.559 129.574 2.30417 1.99828 2.65688

Directly Standardized Rate Estimates

Rate Multiplier = 100000

--------Study Population------- -Reference Population- -----------Standardized Rate----------

Observed Population- Crude Expected Population- Standard 95% Normal

state Events Time Rate Events Time Estimate Error Confidence Limits

1 1216 5664701 21.4663 0.000300 1.00000 29.9615 0.8702 28.2559 31.6671

2 9955 43443610 22.9148 0.000157 1.00000 15.7398 0.1610 15.4242 16.0554

Rate Effect Estimates (Rate Multiplier = 100000)

-----------state----------

North Log

Rhine-Westphalia Rate 95% Lognormal Rate Standard

Cali (NRW) Ratio Confidence Limits Ratio Error Z Pr > |Z|

29.9615 15.7398 **1.9036 1.79206 2.02198** 0.6437 0.0308 20.90 <.0001




* b) Indirect standardized rates;

**proc** **stdrate** data = cali refdata = nrw method = indirect stat = rate(mult = **100000**);

population event = cases total = py;

reference event = cases total = py; strata age/smr stats;

**run**;

Indirectly Standardized Strata Statistics

Rate Multiplier = 100000

----------------------------Study Population---------------------------- ------Reference Population------

Stratum Observed ----Population-Time--- Crude Standard 95% Normal ----Population-Time--- Crude Expected

Index age Events Value Proportion Rate Error Confidence Limits Value Proportion Rate Events

1 1 0 1879110 0.3317 0.000 0.0000 0.000 0.000 8630574 0.1987 0.023 0.435

2 2 74 1886884 0.3331 3.922 0.4559 3.028 4.815 10853391 0.2498 1.225 23.122

3 3 351 1305887 0.2305 26.878 1.4347 24.066 29.690 13425892 0.3090 14.688 191.809

4 4 585 523822 0.0925 111.679 4.6174 102.629 120.729 8725513 0.2008 63.091 330.484

5 5 206 68998 0.0122 298.559 20.8016 257.789 339.330 1808240 0.0416 129.574 89.403

Strata SMR Estimates

Rate Multiplier = 100000

---Study Population-- Reference

Stratum Observed Population- Crude Expected Standard 95% Normal

Index age Events Time Rate Events SMR Error Confidence Limits

1 1 0 1879110 0.023 0.435 0.0000 . . .

2 2 74 1886884 1.225 23.122 3.2004 0.3720 2.4712 3.9295

3 3 351 1305887 14.688 191.809 1.8299 0.0977 1.6385 2.0214

4 4 585 523822 63.091 330.484 1.7701 0.0732 1.6267 1.9136

5 5 206 68998 129.574 89.403 2.3042 0.1605 1.9895 2.6188

Standardized Morbidity/Mortality Ratio

Observed Expected Standard 95% Normal

Events Events SMR Error Confidence Limits Z Pr > |Z|

1216 635.254 **1.9142** 0.0549 **1.8066 2.0218** 16.65 <.0001




* c) Direct standardized rates;

**proc** **stdrate** data = pops refdata = cali method = direct stat = rate(mult = **100000**) effect = ratio;

population group = state event = cases total = PY;reference total = py;

strata age/effect;

**run**;


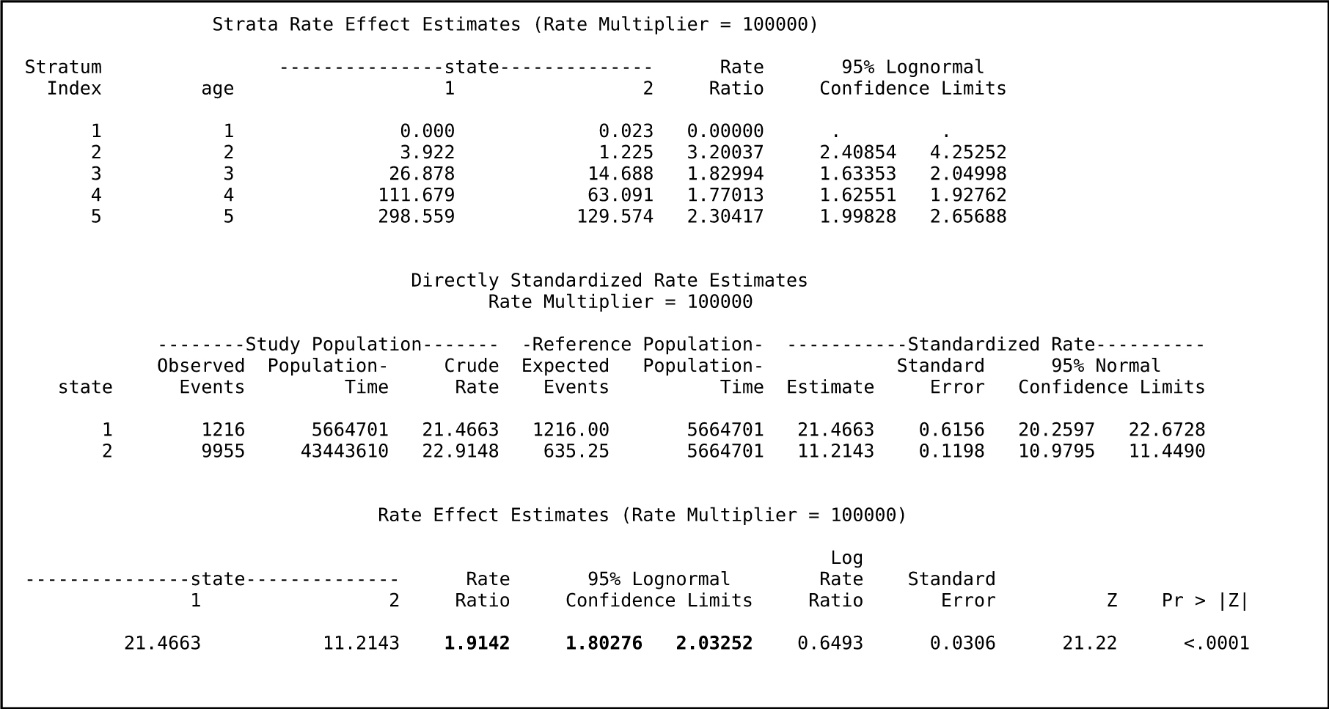

Supplement: Supplementary file 1 — Supplementary file1 (DOCX 21 kb) [file 10654_2025_1349_MOESM1_ESM.docx]
