# Supplementary material for: Indirect standardization: time to eliminate misleading terminology
Source: Eur J Epidemiol. 2026 Jan 24;41(4):527–9. doi: 10.1007/s10654-025-01349-z (PMC13331817; doi:10.1007/s10654-025-01349-z)
Supplement: Supplementary file 2 — Supplementary file2 (DOCX 55 kb) [file 10654_2025_1349_MOESM2_ESM.docx]

**Indirect Standardization: time to eliminate misleading terminology**

Authors: Emilio Gianicolo^1^, Maria Blettner^1^, Andreas Stang^2,3^

Affiliations

^1^ Institut für Medizinische Biometrie, Epidemiologie und Informatik (IMBEI), Universitätsmedizin der Johannes-Gutenberg-Universität, Mainz, Germany

^2^ Institut für Medizinische Informatik. Biometrie und Epidemiologie (IMIBE), Universitätsklinikum Essen, Germany

^3^ North Rhine-Westphalia State Cancer Registry, Bochum, Germany

**Introduction**

Standardization is one of the oldest statistical techniques for the analysis of epidemiological data. It is largely used to fairly compare populations while accounting for the confounding effect of e.g., different age structures.(1, 2) Most textbooks describe two principal approaches to standardizing rates: the so-called *direct* and the so-called *indirect* method. During the 1980’s, however, Miettinen(3) and Rothman(4) argued that there is actually any distinction between the two methods, and in 2002, Rothman explicitly defined the term “indirect standardization” a misnomer because the two approaches are mathematically equivalent.(5)

Both the *direct* and the *indirect* approaches are mathematically identical in that each method computes a weighted sum of age‑specific rates; the only difference lies in the source of the weights: direct standardization uses a set of standard population weights (person-years)(6), whereas *indirect* standardization applies as weights the person-years observed in the exposed population (see supplementary material for the algebra).

As standard population you might choose:

1. an external population, for example the Segi-World population (7) or the Old European standard population; (8)
2. one of the populations you are comparing or their combination.

When the distribution of person years of an exposed population is assumed as the standard, the ratio of the standardized rates is called standardized incidence ratio (SIR) (6) and this way of standardizing is traditionally called the “indirect” standardization (9-11).

In practice, epidemiological text books (12), and major statistical packages – such as SAS® – continue to employ this terminology: *direct standardization* for the calculation of directly standardized rates and *indirect standardization* for the calculation of the standardized incidence ratio (SIR).

The aim of this research letter is to emphasize again the misleading terms “direct” and “indirect” and show with an example how to use SAS® to obtain SIRs while applying the method of the *direct standardization* in place of the traditional *indirect* formulation producing identical results.

**Methods**

We used data on stomach cancer in Cali (Columbia) and North Rhine-Westphalia (NRW, Germany) together with the corresponding population figures for the period 2013–2017 obtained by the International Agency for Research on Cancer.(13)

Analyses were performed in SAS® as described below.

1. We first calculated the SIR for Cali with NRW serving as the reference population, i. e. the conventional indirect method.
2. Next, we derived the identical SIR by the *direct option* of SAS® PROC STDRATE, employing the age-specific person-years from Cali (conceptually the “exposed” group) as the weighting set.

**Results**

In the period 2013-2017 crude incidence rates of 21.5 and 22.9 per 100,000 PY were observed in Cali and NRW respectively (Table 1). The crude rate ratio was 0.94 (95% confidence interval: 0.89-0.99). This result is counterintuitive, as one might expect higher incidence rates in a region of the Global South compared to those of the Global North, given the higher prevalence of risk factors for stomach cancer in the former. However, this pattern is due to the different age structures characterizing the two populations, with prevailing higher percentages of younger in Cali than NRW.

1. The SIR obtained using the “indirect” option equaled 1.91 (Supplementary material, step a).
2. Applying the “direct” option (i. e. using the Cali age‑specific person‑years as the weighting set) produced an identical SIR of 1.91 (Supplementary material, step c).

**Discussion**

In the present contribution we illustrated that in statistical software like in SAS® “direct” and “indirect” options can be used interchangeably to obtain standardized mortality or incidence ratios. Indeed, students of epidemiology are taught that the direct and the indirect method are mathematically equivalent as long as an internal standard is used. Yet, some statistical software packages used by epidemiologists have perpetuated this *misnomer* in their terminology. The time is now ripe, for us teachers of epidemiology and for developers of statistical software packages, to revise this terminology. We propose that, instead of using the labels “direct” and “indirect”, authors simply describe the source of the weights employed in the standardization. This wording clarifies the analytical choice without relying on ambiguous terms.

**Table 1. Number of stomach cancer cases among males, person years and crude incidence rates in Cali (Columbia) and North Rhine-Westphalia (Germany), 2013-2017.**

| Age group  (years) | Cali | | |  | North Rhine-Westphalia | | |
| --- | --- | --- | --- | --- | --- | --- | --- |
|  | Number of stomach cancer cases | Person-years | Crude rates per 100.000 person-years |  | Number of stomach cancer cases | Person-years | Crude rates per 100.000 person-years |
| 0-19 | 0 | 1,879,110 | 0.0 |  | 2 | 8,630,574 | 0.0 |
| 20-39 | 74 | 1,886,884 | 3.9 |  | 133 | 10,853,391 | 1.2 |
| 40-59 | 351 | 1,305,887 | 26.9 |  | 1,972 | 13,425,892 | 14.7 |
| 60-79 | 585 | 523,822 | 111.7 |  | 5,505 | 8,725,513 | 63.1 |
| ≥80 | 206 | 68,998 | 298.6 |  | 2,343 | 1,808,240 | 129.6 |
| **Total** | **1,216** | **5,664,701** | **21.5** |  | **9,955** | **43,443,610** | **22.9** |

**Supplementary material**

**Algebra**

Given two populations A and B, the ratio of two standardized rates $R^{A*}$and $R^{B*}$ can be expressed as:

$${RR}_{std}=\frac{R^{A*}}{R^{B*}}=\frac{\sum_{i=1}^{k} {PY}_{i}^{*}\times R_{i}^{A}}{\sum_{i=1}^{k} {PY}_{i}^{*}\times R_{i}^{B}}$$

where $i=1 to k$ indicates the age group;

${PY}_{i}^{*}$ represents the distribution of the person years of a standard population;

$R_{i}^{A}$ and $R_{i}^{B}$represent age specific rates for a given outcome $m_{i}$for the populations A ($R_{i}^{A}=\frac{m_{i}^{A}}{{PY}_{i}^{A}}$) and B ($R_{i}^{A}=\frac{m_{i}^{A}}{{PY}_{i}^{A}}$).

**SAS program.**

**proc** **format**;

value age

**1**="0-19 years" **2**="20-39" **3**="40-59" **4**="60-79" **5**="=>80";

value state

**1**="Cali" **2**="North Rhine-Westphalia (NRW)"; **run**;

**data** pops;

attrib

state label="State"

age label="Age-class"

cases label="Number of cases"

py label="Person-years"

;input state age cases py;

cards;

1 1 0 1879110

1 2 74 1886884

1 3 351 1305887

1 4 585 523822

1 5 206 68998

2 1 2 8630574

2 2 133 10853391

2 3 1972 13425892

2 4 5505 8725513

2 5 2343 1808240

;

**run**;

**data** cali nrw;

set pops;

if state=**1** then output cali;

if state=**2** then output nrw;

**run**;

* a) Standardized rates obtained using the “indirect” option;

**proc** **stdrate** data=cali refdata=nrw method=indirect stat=rate(mult=**100000**);

population event=cases total=py;

reference event=cases total=py; strata age/smr stats;

**run**;

Indirectly Standardized Strata Statistics

Rate Multiplier = 100000

----------------------------Study Population---------------------------- ------Reference Population------

Stratum Observed ----Population-Time--- Crude Standard 95% Normal ----Population-Time--- Crude Expected

Index age Events Value Proportion Rate Error Confidence Limits Value Proportion Rate Events

1 1 0 1879110 0.3317 0.000 0.0000 0.000 0.000 8630574 0.1987 0.023 0.435

2 2 74 1886884 0.3331 3.922 0.4559 3.028 4.815 10853391 0.2498 1.225 23.122

3 3 351 1305887 0.2305 26.878 1.4347 24.066 29.690 13425892 0.3090 14.688 191.809

4 4 585 523822 0.0925 111.679 4.6174 102.629 120.729 8725513 0.2008 63.091 330.484

5 5 206 68998 0.0122 298.559 20.8016 257.789 339.330 1808240 0.0416 129.574 89.403

Strata SMR Estimates

Rate Multiplier = 100000

---Study Population-- Reference

Stratum Observed Population- Crude Expected Standard 95% Normal

Index age Events Time Rate Events SMR Error Confidence Limits

1 1 0 1879110 0.023 0.435 0.0000 . . .

2 2 74 1886884 1.225 23.122 3.2004 0.3720 2.4712 3.9295

3 3 351 1305887 14.688 191.809 1.8299 0.0977 1.6385 2.0214

4 4 585 523822 63.091 330.484 1.7701 0.0732 1.6267 1.9136

5 5 206 68998 129.574 89.403 2.3042 0.1605 1.9895 2.6188

Standardized Morbidity/Mortality Ratio

Observed Expected Standard 95% Normal

Events Events SMR Error Confidence Limits Z Pr > |Z|

1216 635.254 **1.9142** 0.0549 **1.8066 2.0218** 16.65 <.0001

* b) obtained using the “direct” option;

**proc** **stdrate** data=pops refdata=cali method=direct stat=rate(mult=**100000**) effect=ratio;

population group=state event=cases total=PY;reference total=py;

strata age/effect;

**run**;

Strata Rate Effect Estimates (Rate Multiplier = 100000)

Stratum ---------------state-------------- Rate 95% Lognormal

Index age 1 2 Ratio Confidence Limits

1 1 0.000 0.023 0.00000 . .

2 2 3.922 1.225 3.20037 2.40854 4.25252

3 3 26.878 14.688 1.82994 1.63353 2.04998

4 4 111.679 63.091 1.77013 1.62551 1.92762

5 5 298.559 129.574 2.30417 1.99828 2.65688

Directly Standardized Rate Estimates

Rate Multiplier = 100000

--------Study Population------- -Reference Population- -----------Standardized Rate----------

Observed Population- Crude Expected Population- Standard 95% Normal

state Events Time Rate Events Time Estimate Error Confidence Limits

1 1216 5664701 21.4663 1216.00 5664701 21.4663 0.6156 20.2597 22.6728

2 9955 43443610 22.9148 635.25 5664701 11.2143 0.1198 10.9795 11.4490

Rate Effect Estimates (Rate Multiplier = 100000)

Log

---------------state-------------- Rate 95% Lognormal Rate Standard

1 2 Ratio Confidence Limits Ratio Error Z Pr > |Z|

21.4663 11.2143 **1.9142 1.80276 2.03252** 0.6493 0.0306 21.22 <.0001

Please note that confidence intervals are not identical, as they are based on different distributions.

**Competing Interests**

The authors have no relevant financial or non-financial interests to disclose.

**Bibliography**

1. Feinleib M, Zarate A. Reconsidering age adjustment procedures; workshop proceedings. Hyattsville, MD. 1992.

2. Keiding N. The method of expected number of deaths, 1786-1886-1986. Int Stat Rev. 1987;55(1):1-20.

3. Miettinen OS. Theoretical Epidemiology: Principles of Occurrence Research in Medicine. New York: John Wiley and Sons, Inc.,; 1985.

4. Rothman K. Modern Epidemiology 1st Edition: Little Brown & Co.; 1986.

5. Rothman K. Epidemiology An Introduction 1st edition: Oxford University Press Inc; 2002.

6. Lash T, VanderWeele T, Haneuse S, Rothman K. Modern Epidemiology: Lippincott Williams and Wilkins; 2021 4th Edition.

7. Segi M. Cancer mortality for selected sites in 24 countries, no. 4, 1950-57. Sendai, Japan: Tohoku University of Medicine; 1960.

8. Doll R, Cook P. Summarizing indices for comparison of cancer incidence data. Int J Cancer. 1967;2(3):269-79. doi:10.1002/ijc.2910020310

9. Hennekens C, Buring J. Epidemiology in Medicine: Lippincott Williams & Wilkins; 1987.

10. Szklo M, Nieto F. Epidemiology: Beyond the Basics: Publisher: Jones & Bartlett Learning; 2004.

11. Gordis L. Epidemiology: Saunders; 2004 2nd Edition.

12. Webb P, Bain C, Page A. Essential Epidemiology: Cambridge University Press; 2024 5th Edition.

13. Bray F, Colombet M, Aitken J, et al. Cancer Incidence in Five Continents. Lyon: International Agency for Research on Cancer. 2023.
